# Effective cellular internalization of silica-coated CdSe quantum dots for high contrast cancer imaging and labelling applications

**DOI:** 10.1186/s12645-014-0001-y

**Published:** 2014-06-27

**Authors:** Muthunayagam Vibin, Ramachandran Vinayakan, Annie John, Francis Boniface Fernandez, Annie Abraham

**Affiliations:** Department of Biochemistry, University of Kerala, Kariavattom campus, 695581 Thiruvananthapuram, Kerala India; Photosciences and Photonics, National Institute for Interdisciplinary Science and Technology (CSIR), Thiruvananthapuram, Kerala India; TEM Laboratory, Sree Chitra Tirunal Institute of Medical Sciences and Technology, Biomedical Technology Wing, Thiruvananthapuram, Kerala India

**Keywords:** Stem cells, Cellular imaging, Fluorescence, Internalization, Quantum dots, Ultrathin

## Abstract

The possibility of developing novel contrast imaging agents for cancer cellular labelling and fluorescence imaging applications were explored using silica-coated cadmium selenide (CdSe) quantum dots (QDs). The time dependent cellular internalization efficiency study was carried out using Inductively Coupled Plasma-Optical Emission Spectroscopy (ICP-OES) and Confocal Laser Scanning Microscopy (cLSM) after exposing QDs to stem cells and cancer cells. The strong fluorescence from the cytoplasm confirmed that the QDs were efficiently internalized by the cells. The internalization maxima were observed at the fourth hour of incubation in both stem and cancer cells. Further, the in vitro fluorescence imaging as well as localization study of QDs were performed in various cells. Moreover, high contrast in vivo tumor imaging efficiency of silica-coated CdSe QDs was performed in ultrathin sections of tumor mice, and the results confirmed its effective role in cellular imaging and labelling in cancer and other diseases.

## Background

The development of highly sensitive and specific biological probes which lack the intrinsic limitations of organic dyes and fluorescent proteins is focus of many areas of research like molecular and cellular biology [1,2] and medical diagnostics [3–6]. The conventional organic fluorescent tags mainly suffer from low photo stability, poor quantum yield under biological conditions and interference from auto fluorescence. The exceptional photophysical properties of quantum dots (QDs), particularly photostability and emission as a function of size, make them superior to organic dyes for biological applications. These properties have opened new possibilities for advanced molecular and cellular imaging as well as for ultrasensitive bioassays and diagnostics [7–10]. Besides, QDs are better labelling agents in long-term imaging such as fluorescence marking of transport processes in cells and in tracking the path of single membrane-bound molecules [11,12].

Although imaging of fixed cells is useful and sufficient for many applications, live cell microscopy is ideal for visualizing cellular processes, which is considerably more difficult. It has been shown that many cell types naturally engulf QDs through a non-specific uptake mechanism. This mechanism was used to track the migration of breast tumor cells on a substrate coated with red emitting QDs; the fluorescence within the cells were increased due to the uptake of QDs, leaving behind a dark path [13–15].

Many reports highlight the use of QDs as a fluorescent probe to visualize the biological processes both in vitro and in vivo [16–18]. With proper surface functionalization using peptides, proteins or antibodies, QDs shows specificity in targeting and imaging tumors in living subjects. The fluorescence imaging can easily be achieved by monitoring the stable and strong fluorescence from the QDs [19]; Law et al. [20]. These studies confirm that QDs have opened up a new avenue for investigating biomolecular processes inside the cells.

The inherent hydrophobicity of QDs can be overcome by various approaches; among these overcoating with silica is most preferred method [21–24]. Besides, the cytocompatibility of CdSe QDs on overcoating with silica has been well established by many groups [25–27]. This is based on the observation that the core constituents viz., cadmium and selenium ions are well encapsulated with in the silica shell preventing surface oxidation, even in the biological media. Thus a silica shell plays dual role i.e., it makes QDs dispersible in aqueous media and it eliminates the toxicity. A detailed investigation of the cytocompatibility of silanised QDs was reported by our group [28]. By time and concentration dependent studies we observed that the internalized silica-coated CdSe QDs were non-toxic proving the cytocompatibility even at higher concentrations and longer incubation periods. We have also reported the non-specific cellular uptake and subcellular localization of silica-coated CdSe quantum dots [29].

Several research groups have described the use of QDs for sensitive bioassays and cellular imaging in vitro and in vivo [30–34]. But many aspects of this approach need to be further optimized particularly for in vivo applications. Our preliminary in vitro investigations proved the cytocompatibility and stability of silica-coated CdSe QDs under biological conditions [29]. The objective of this study was to demonstrate that the silica-coated CdSe QDs can be used as labelling agents for long-life cellular imaging, cancer cellular imaging using cLSM and fluorescence microscopy. We use Inductively Coupled Plasma-Optical Emission (ICP-OES) Spectroscopy to quantify the in vitro cellular internalization efficiency of silica-coated CdSe QDs in a couple of cell models, New Zealand rabbit adipose derived mesenchymal stem cells (RADMSCs) and Human cervical cancer cells (HeLa). Imaging using Confocal microscopy is also performed to obtain a visual image of the QDs internalised by cells after incubation for a time interval, assessed from the results of ICP-OES studies. Simple fluorescence imaging and localization of the silica-coated CdSe QDs studies using fluorescent microscopy after staining with 4,6-diamidino-2-phenylindole (DAPI) have also been carried out in RADMSCs and HeLa cells. The high cellular internalisation efficiency as well as the high contrast confocal images of tumor sections obtained indicates the role of silica-coated CdSe QDs in imaging and labelling of cancer cells and other diseases.

## Methods

### Chemicals

Chemicals for QD synthesis and silica overcoating such as trioctylphosphine oxide (TOPO), trioctylphosphine, cadmium oxide (CdO), selenium powder, dodecylamine, igepal and aminopropyl silane (APS) were purchased from Sigma–Aldrich and tetradecylphosphonic acid (TDPA) from Alfa Aesar and used as such without further purification.

### Synthesis and characterization of silica-coated CdSe QDs

QDs were synthesized by following a reported procedure [35,36]. Cadmium oxide (0.067 g, 0.52 mmol), dodecylamine (3.8 g, 20.72 mmol), trioctylphosphine oxide (2.7 g, 6.9 mmol) and tetradecylphosphonic acid (0.40 g, 1.44 mmol) were heated to 300°C under vacuum, until CdO dissolved completely to produce an optically clear solution. At this temperature, an injection mixture containing trioctylphosphine (5.2 mL) and Se (0.083 mmol) was introduced. After desired crystal growth, the reaction was arrested by reducing the reaction temperature down to ambient conditions. The QDs were purified by reprecipitation with methanol and redispersed in dry chloroform for silica overcoating.

For overcoating CdSe QDs with silica, we have modified the reported procedure [18, 24] as follows: A mixture of TOPO capped CdSe QDs in chloroform (400 μL) and APS (0.075 g, 0.36 mM) was vortexed for 30 min, in an inert atmosphere. This mixture was added to Igepal CO-520 (1.3 mL) in cyclohexane (10 mL) and stirred for 30 min under dry conditions. Ammonia solution (150 μL, 33 wt.%) was added drop wise and the stirring was continued for 1 day. The silica-coated QDs were purified by washing repeatedly with dry chloroform and redispersed in PBS buffer (pH 7.3). Dots were stored in the dark at room temperature and the emission stability was investigated as a function of time, pH and ionic strength of the medium.

The electronic absorption spectra were recorded on a Shimadzu UV-3101 scanning spectrophotometer; emission spectra were collected using SPEX-Fluorolog F112X spectrofluorimeter. For HRTEM studies, a drop of nanoparticle solution was placed on a carbon coated Cu grid and the solvent was allowed to evaporate. Specimens were imaged on a FEI Tecnai G2 S-TWIN 300 kV high resolution transmission electron microscope.

### Materials and animals

For cellular imaging experiments, all chemicals and reagents used were purchased from Sigma–Aldrich, USA. The New Zealand rabbit adipose tissue-derived mesenchymal stem cells (RADMSCs) and Human cervical cancer cells (HeLa) were maintained at 37°C and 5% CO_2_ in Dulbecco’s Modified Eagle’s Medium (DMEM) supplemented with 10% fetal bovine serum (FBS) and antibiotics.

The animal models used for the tumor imaging study (Normal male, Swiss albino mice of 6 weeks old, weighing 20–25 g) were from the animal house facility, Department of Biochemistry, University of Kerala. For maintaining the experimental animals, the institutional ethical guidelines were absolutely followed as per Committee for the Purpose of CPCSEA rules [Sanction No: IAEC-KU-9/06-07/BC-AA (8) (ii)], Government of India. All animal experiments were performed in triplicate.

#### Solid tumor development

The solid tumor model was established by subcutaneous injection of DLA cells (1 × 10^6^ cells/animal) into the back right hind limb of mice [37]. The mice were subjected to imaging studies when the tumor volume reached 200–450 mm^3^ (3–4 weeks after inoculation).

#### Percentage of cellular internalization of silica-coated CdSe QDs – ICP-OES

For silica-coated CdSe QDs internalization studies, HeLa and RADMSCs were seeded 1 × 10^6^ cells/well in 6-well plates and incubated overnight to allow for cell attachment. After the overnight incubation, cell medium was removed and replaced with 1 mL of DMEM medium. Cells were serum starved for 30 min and fresh medium containing the silica-coated CdSe QDs was added. Cells were incubated for different time interval (0, 1, 2, 3, 4 and 5 h) in the presence of silica-coated CdSe QDs. At each time point, cell supernatant was removed and cells were washed three times with PBS. Samples were digested in 500 μL of nitric acid for 30 min at 70°C and diluted in 3 mL of double distilled water. Quantum dots internalization was quantified using ICP-OES (Optima 5300 DV instrument, Perkin Elmer). All experiments were performed in triplicate.

#### Cellular internalization studies of silica-coated CdSe QDs – cLSM

To study the cellular internalization efficiency of the silica-coated CdSe QDs in vitro, RADMSCs and HeLa cells were cultured on coverslips and the medium was changed on every 2 days, till 85% cell confluence was achieved. Then the cells were incubated at 37°C with 5% CO_2_ for 2 and 4 h after adding with silica-coated CdSe QD solution (100 nM) in PBS. After incubation, the coverslips were taken out, rinsed thrice at 37°C using pre-heated PBS, fixed with 3.7% paraformaldehyde and fluorescence images were observed in a confocal laser scanning microscope (cLSM). An argon laser (488 nm) was used for QD excitation under confocal fluorescence microscope (Carl Zeiss LSM 510 META). All experiments were performed in triplicate.

#### Cellular imaging and localization study using silica-coated CdSe QDs – Fluorescence microscopy

For cellular imaging experiment, stem cells (RADMSC) and cancer cells (HeLa) were used. RADMSCs and HeLa were cultured on coverslips, and the medium was changed every 2 days, until 85% cell confluence was achieved. Then the cells were incubated at 37°C with 5% CO_2_ for 4 h after adding with silica-coated CdSe QD solution (100 nM in PBS). Then, silica-coated CdSe QD solution (100 nM) was added to the cells. After incubation, the coverslips were taken out, rinsed thrice at 37°C using pre-heated PBS, fixed with 3.7% paraformaldehyde, after staining with 4,6-diamidino-2-phenylindole (DAPI) and fluorescence cellular images were observed using a DM 6000 fluorescence microscope (Leica, Germany, 20x objective, equipped with DFC 300 FX digital camera).

In the case of fluorescence imaging of cancer cells - Daltons Lymphoma Ascites (DLA) cells, were procured from Amala Cancer Research Centre, Thrissur, India and maintained in the peritoneal cavity of mice. Approximately 2 weeks were taken for the development of tumor in the peritoneal cavity and matured cells were aspirated from the peritoneum, washed with PBS and seeded on coverslips. Then the cells were incubated at 37°C with 5% CO_2_ for 2 and 4 h after adding with silica-coated CdSe QD solution (100 nM) in PBS. After incubation, the coverslips were taken out, rinsed thrice at 37°C using pre-heated PBS, fixed with 3.7% paraformaldehyde and fluorescence images were observed in a DM 6000 fluorescence microscope (Leica, Germany, 20x objective, equipped with DFC 300 FX digital camera).

#### Fluorescence imaging of ultrathin sections of tumor– cLSM

Tumor mice were anesthetized by intra-abdominal injection of 3% pentobarbital sodium (45 mg/kg). QD and QD-Ab probe were (10 nM) injected at the dosage of 20 ml/kg into the tail vein. After the injection, the mice were put in a dark chamber for 4 h, and then tumor region were stripped from the mice. Then tumor tissues was fixed and sectioned by ultramicrotome (LKB; Bromma-2088-Ultratome®V, Sweden), to obtain 100 nm ultrathin sections of tumor. For fluorescence imaging, these ultrathin tissue sections were directly examined with a cLSM. The ultrathin sections were stained with toludine blue and observed under a light microscope (Leica-DMIL, Germany).

## Results and discussion

**Figure 1:**
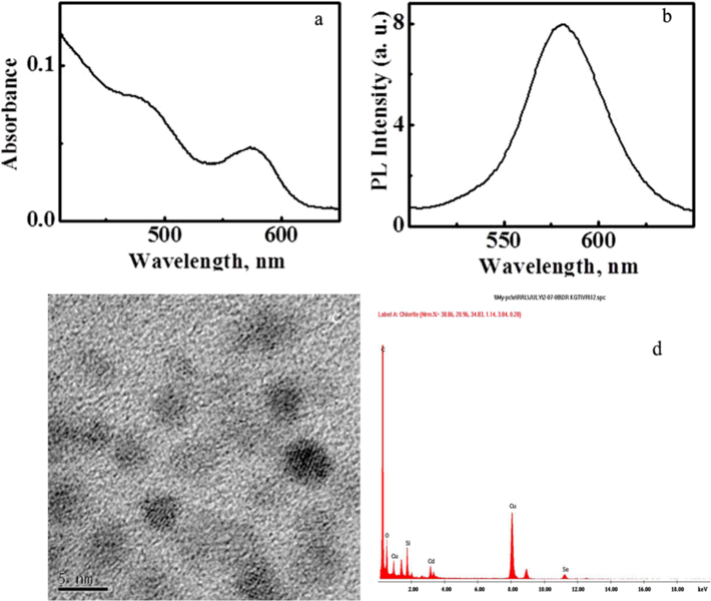
**Characterization of colloidal properties of silica-coated CdSe QDs.** Absorption **(a)** and emission **(b)** spectra in PBS buffer (pH 7.3); HRTEM image **(c)**. EDS spectrum **(d)** of silica-coated CdSe QDs.

Trioctylphosphine oxide capped CdSe QDs were synthesised and made water soluble by silanisation. The details of QDs synthesis, silanisation and the evaluation of colloidal properties of QDs have been published elsewhere in detail [29]. The silica-coated CdSe QDs obtained showed a greenish yellow emission in PBS buffer (pH 7.3). The size homogeneity was ensured using high resolution transmission electron microscope (HRTEM), and colloidal properties of QDs were depicted in Figure 1. The QDs were found to be readily soluble in aqueous medium, stable and luminescing under biological conditions.

**Figure 2:**
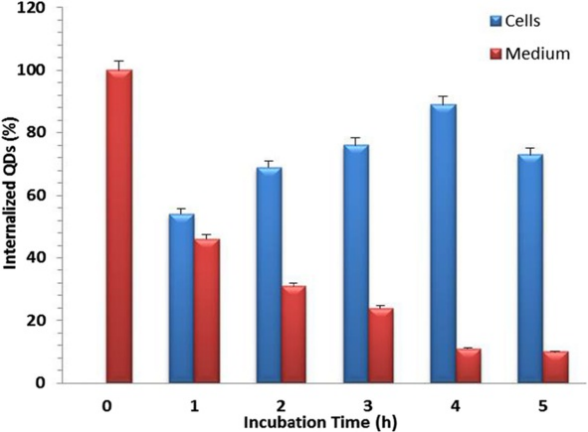
**Percentage of cellular internalixzation of silica-coated CdSe QDs in stem cell (RADMSC) using ICP-OES.** The peak internalization was observed at 4 h after the QD exposure.

**Figure 3:**
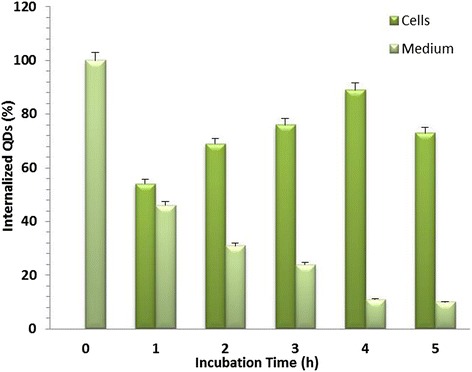
**Percentage of cellular internalization of silica-coated CsdSe QDs in cancer cells (HeLa) using ICP-OES.** The peak internalization was observed at 4 h after the QD exposure.

The cytocompatibility of cadmium chalcogenides based QDs are of primary attention when there biological applications are concerned. The toxicological effect is attributed to the leaching out of core materials, particularly cadmium ions into the cell media [38,39]. The non-toxicity of the QDs used in our studies is ensured by generating a CdSe/Silica core shell structure preventing any leakage of core materials. Also, the silica shell ensures aqueous solubility which is essential for biological studies and applications [28]. One of the emerging applications of QDs is cellular labelling and imaging. In the current experiment, we have used a couple of cell models-HeLa cells and RADMSCs, to monitor the in vitro cellular internalisation efficiency of silica-coated QDs as a function of time. The results obtained from the ICP-OES studies undoubtfully prove the high internalisation efficiency and cytocompatibility of silica-coated CdSe QDs. These observations confirm their potential applications in cellular imaging. This aspect was further confirmed by confocal laser scanning microscopy (cLSM) studies. For this, cells were incubated with QDs for 1, 2, 3, 4 and 5 h and then washed to remove any unbound QDs. Percentage of cellular internalization of silica-coated CdSe QDs were studied by using Inductively Coupled Plasma-Optical Emission Spectroscopy (ICP-OES). ICP-OES was performed utilizing an Optima 5300 DV instrument (Perkin Elmer). ICP-OES was used to measure the cadmium content to indicate the concentration of QDs in cells collected at predetermined time intervals (0–5 h). The ICP-OES instrument was initialized, optimized and standardized using the manufacturer’s recommendations. Standardization was performed using multielement standards (Perkin Elmer Life and Analytical Sciences). The result obtained from the ICP-OES studies is shown in Figures 2 & 3, where the percentage of internalised QDs is plotted as a function of incubation time. Results showed that the maximum internalization observed at 4 h in both stem and cancer cells after the QD exposure. Overall internalization efficiency was found to be significant high in cancer cells when compared to stem cells (Figures 2 & 3).

**Figure 4:**
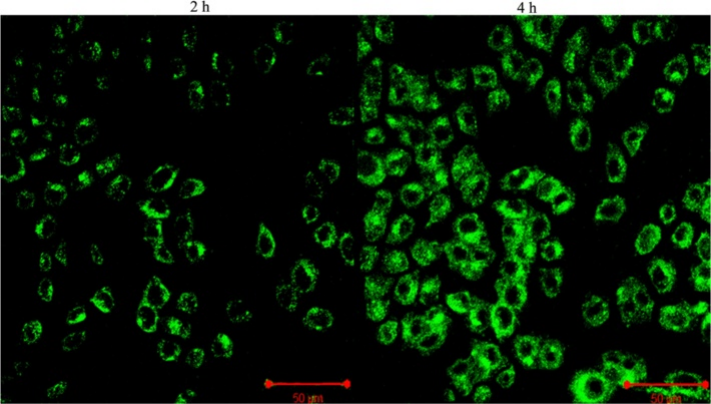
**Cellular internalization efficiency of silica-coated CdSe QDs in cancer using cLSM study; QDs were incubated in presence of HeLa cells.** The cLSM micrographs confirmed the peak internalization of QDs at 4 h.

**Figure 5:**
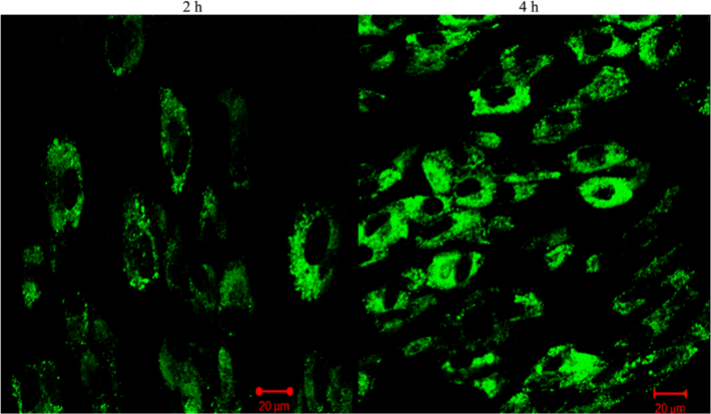
**Cellular internalization efficiency of silica-coated CdSe QDs in stem cells using cLSM study; QDs were incubated in presence of RADMS cells.** The cLSM micrographs confirmed the peak internalization of QDs at 4 h.

The cellular internalization efficiency of silica-coated CdSe QDs was monitored by confocal laser scanning microscopy (cLSM). For this, cells were incubated with QDs for the predetermined time intervals and then washed to remove any unbound QDs. Interestingly, we were able to observe a uniform fluorescence with high contrast in most of the cells, when examined under cLSM (Figures 4 & 5). The peak internalization time (4 h) was again confirmed by the images obtained from cLSM analysis. cLSM imaging were performed in all time intervals (1, 2, 3, 4 and 5 h), and images for 2 and 4 h are showing in Figures 4 & 5. The fluorescence signal was seen in cytoplasm as well as in the vicinity of nucleus, in case of both cell models under study. These results confirmed the significant internalization of QDs into cytoplasmic vesicles. The fluorescence obtained within the cells is attributed to the trapping of QDs in the endocytic intracellular vesicles. These observations are in agreement with previous reports demonstrating the uptake of QD conjugates, which was mediated by endocytosis [40]. Jaiswal and his co-workers also reported a similar mechanism of QDs uptake, and the fluorescence observed from cells was attributed to the internalization of QDs by endocytosis [13]. Thus internalized silica-coated CdSe QDs were non-toxic during this time span at concentrations needed for uptake study in these cells. Cytocompatibility of these fluorescent materials were reported earlier by our previous report [28], indicating that the silica layer makes the QD dispersible in aqueous media and also prevents the leakage of toxic cadmium and selenium ions. The silica-coated CdSe QDs were found to be efficiently internalized by cancer cells and stem cells in vitro, thereby highlighting their potential to be used as a non-toxic optical probe for biomedical diagnostics as well as cellular labelling applications. To the best of our knowledge, this is the first report of using silica-coated CdSe QDs for the cellular imaging, cancer imaging and internalization studies.

**Figure 6:**
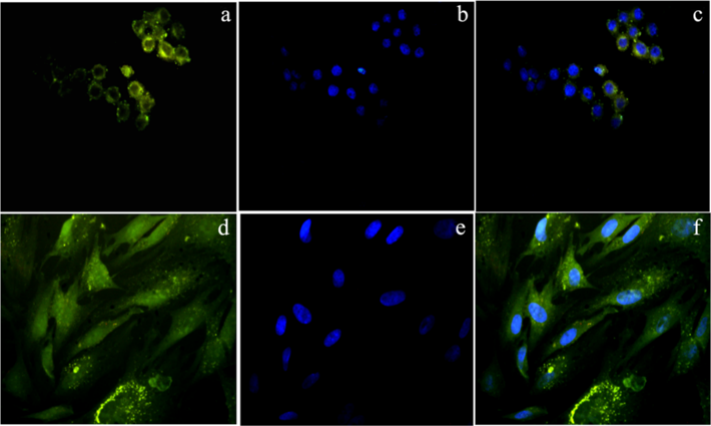
**Cellular imaging-fluorescence microscopy: (a & d) HeLa and RADMSC cells showed the efficient cellular internalization of QDs in the cytoplasm and nearby nucleus [yellowish green fluorescene]; (b & e) Nucleus were counterstained blue with DAPI [blue flurescene]; (c & f) Overlay of QDs and DAPI fluorescene images.**

Further, we have applied these QDs for different cellular imaging applications and localization studies by growing the cancer cells (HeLa) and stem cells (RADMSC) for 4 h in the presence of QDs. Before the cellular imaging experiment, the cell viability was studied systematically using cell viability assays, and the cell proliferation assay data showed the absence of cell death (in comparison to control experiments), confirming that the core material is well protected inside the silica shell (data not shown) and these observations were published elsewhere [28]. In 2009, Han’s group reported the CdSeS QD/SiO_2_ nanoparticles showing good resistance to photobleaching and low cytotoxicity, which facilitates their use in live cell imaging [27]. Our cellular imaging results showed that silica-coated CdSe QDs were significantly internalized into cytoplasmic vesicles and produce the good fluorescence in and around the cells (both stem and cancer cells) (Figure 6). Generally, we found that the silica-coated CdSe QDs show much higher cellular internalization efficiency, possibly because of cytocompatibility of silica shell.

**Figure 7:**
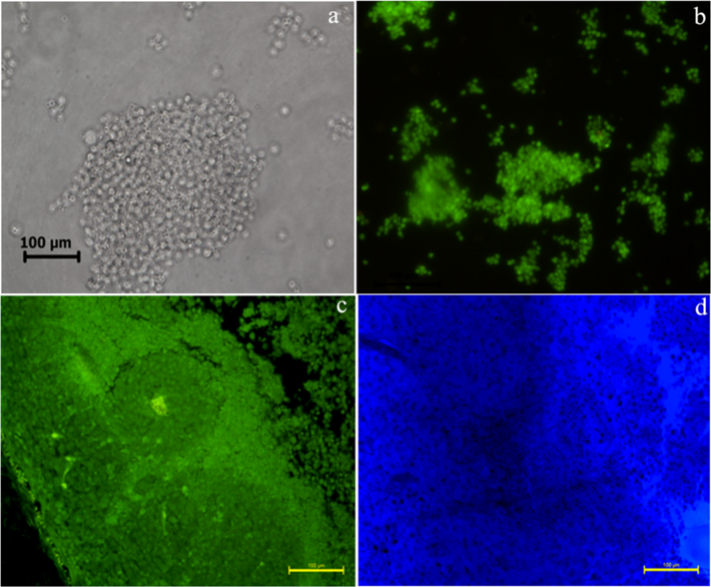
**Fluorescene imaging of tumor sections using silica-coated CdSe Qds.** Phase contrast microscopic image of DLA cells **(a)**. Fluoscene microscope image of DLA cells **(b)**. Fluoscene microscope image of ultra thin section of solid tumor **(c)**. Light microscopic image of toludine blue stained ultrathin section of tumor **(d)**.

Interestingly, in vivo fluorescence based tumor imaging results showed better localisation of QDs in the tumor site (Figure 7). Evident from the results shown in Figure 7b & c, the high contrast fluorescence signal appeared in the DLA cells in vitro and in vivo (ultra-thin section), and Figure 7a shows morphology of DLA cells using phase contrast microscopy. Ultrathin sections of tumor tissue exhibited strong fluorescence which specifies the tumor-targeted imaging efficiency of QDs in living mice (Figure 7c). The light microscopic image of toludine blue stained ultrathin tumor sections showing the presence of growing DLA cells in the tumor (Figure 7d). With these excellent properties and efficient internalization efficiency, silica-coated CdSe QDs might become a highly promising probe for biological applications. These results establish silica-coated CdSe QDs as an extremely useful tools for molecular imaging, cell tracking and labelling to study the cell division and every cellular events especially metastasis of cancer and other diseases.

## Conclusions

The in vitro cellular internalization efficiency of silica-coated CdSe QDs is systematically followed in this report. Cytocompatibility assessment experiments based on various assays showed that the silanised QDs were non-toxic, aqueous soluble and showed stable fluorescence under biological conditions. The ICP-OES measurements in stem cells (RADMSC) and in cancer cells (HeLa) confirmed that silica-coated CdSe QDs have excellent effective internalization efficiency and the peak concentration was observed after 4 hours. In addition, the high contrast images obtained in confocal laser scanning microscopy (cLSM) from the in vitro cellular imaging study on stem cells and cancer cells and in vivo tumor imaging studies further confirmed that these QDs are highly useful for cellular imaging and labelling applications using their relatively stable fluorescence emission under biological conditions. Overall, this study implies that silica-coated CdSe QDs could be used as labelling and imaging agents for cancer cellular imaging and cell tracking applications for the study of cancer and other diseases.
